# Short-term airborne particulate matter exposure alters the epigenetic landscape of human genes associated with the mitogen-activated protein kinase network: a cross-sectional study

**DOI:** 10.1186/1476-069X-13-94

**Published:** 2014-11-13

**Authors:** Juan Jose Carmona, Tamar Sofer, John Hutchinson, Laura Cantone, Brent Coull, Arnab Maity, Pantel Vokonas, Xihong Lin, Joel Schwartz, Andrea A Baccarelli

**Affiliations:** Laboratory of Human Environmental Epigenetics, Department of Environmental Health, Harvard School of Public Health, Boston, MA USA; Exposure, Epidemiology, and Risk Program, Department of Environmental Health, Harvard School of Public Health, Boston, MA USA; Program in Quantitative Genomics, Department of Biostatistics, Harvard School of Public Health, Boston, MA USA; Center for Health Bioinformatics, Harvard School of Public Health, Boston, MA USA; Department of Clinical Sciences and Community Health, Università degli Studi di Milano, Milan, Italy; Department of Statistics, North Carolina State University, Raleigh, NC USA; VA Normative Aging Study, Veterans Affairs Boston Healthcare System and the Department of Medicine, Boston University School of Medicine, Boston, Massachusetts USA

## Abstract

**Background:**

Exposure to air particulate matter is known to elevate blood biomarkers of inflammation and to increase cardiopulmonary morbidity and mortality. Major components of airborne particulate matter typically include black carbon from traffic and sulfates from coal-burning power plants. DNA methylation is thought to be sensitive to these environmental toxins and possibly mediate environmental effects on clinical outcomes *via* regulation of gene networks. The underlying mechanisms may include epigenetic modulation of major inflammatory pathways, yet the details remain unclear.

**Methods:**

We sought to elucidate how short-term exposure to air pollution components, singly and/or in combination, alter blood DNA methylation in certain inflammation-associated gene networks, MAPK and NF-κB, which may transmit the environmental signal(s) and influence the inflammatory pathway *in vivo*. To this end, we utilized a custom-integrated workflow—molecular processing, pollution surveillance, biostatical analysis, and bioinformatic visualization—to map novel human (epi)gene pathway-environment interactions.

**Results:**

Specifically, out of 84 MAPK pathway genes considered, we identified 11 whose DNA methylation status was highly associated with black carbon exposure, after adjusting for potential confounders—age, sulfate exposure, smoking, blood cell composition, and blood pressure. Moreover, after adjusting for these confounders, multi-pollutant analysis of synergistic DNA methylations significantly associated with sulfate and BC exposures yielded 14 MAPK genes. No associations were found with the NF-κB pathway.

**Conclusion:**

Exposure to short-term air pollution components thus resulted in quantifiable epigenetic changes in the promoter areas of MAPK pathway genes. Bioinformatic mapping of single- *vs.* multi-exposure-associated epigenetic changes suggests that these alterations might affect biological pathways in nuanced ways that are not simply additive or fully predictable *via* individual-level exposure assessments.

**Electronic supplementary material:**

The online version of this article (doi:10.1186/1476-069X-13-94) contains supplementary material, which is available to authorized users.

## Background

Exposure to air particulate matter (PM) is well known to augment oxidative stress in exposed individuals, and it has been consistently linked to reduced lung function as well as hospitalization and mortality for various cardiopulmonary diseases [1]. Indeed, ambient air pollution is a critical public health concern in US cities and worldwide, accounting for a staggering ~3.7 million premature deaths globally in 2012, according to a World Health Organization estimate [2, 3]. In highly PM-exposed individuals, blood leukocyte gene expression profiling has revealed responses related to worsened systemic oxidative stress and inflammation, which exacerbate aggravation of the airways and disease outcomes [4], yet the underlying mechanisms remain unclear.

Recent interest has focused on examining different components of air pollution particles to assess their relative toxicity *in vivo*. For example, black carbon (BC), a component of PM commonly used as a marker of traffic particles, has been reported to be more strongly associated with increased blood pressure in the elderly compared to other pollutants [5]. But sulfates—*i.e.*, PM components from industrial emissions—had comparable effect as BC on dilation of the brachial artery [6]. Similarly, traffic pollutants are often more commonly cited as associated with asthma [7]. However, PM with an aerodynamic diameter <2.5 μm (PM_2.5_) had stronger associations than BC in some studies [7, 8], suggesting that PM from sources other than traffic has stronger effects on asthma. Interestingly, sulfates have also been associated with asthma phenotypes [9]. While there are many components of particles in PM, BC as a general index of traffic-derived particles and sulfate as a marker of secondary particles, such as coal-burning power plants, are reasonable surrogates for two of its more important sources. At present, however, questions remain about how best to model these two pollutants, individually and/or in combination, in order to understand their effects on human populations [10].

DNA methylomics is an emerging field that can provide unique opportunities to study source- and component-specific biological effects from PM-related exposures *in vivo*[11–14]. Many nuclear-encoded genes have been shown to undergo exposure-related changes in their DNA methylation status, an epigenetic mechanism highly sensitive to chemical components found in PM [13–17]. DNA methylation, the best understood of the epigenetic mechanisms, is the covalent addition of methyl groups to cytosine to form 5-methyl-cytosine (5mC). Methylation of promoter regions and other regulatory sequences usually tends to repress gene expression, although examples leading to gene activation exist; consequently, DNA methylation is now recognized as an important regulator of transcription [11, 18, 19]. Since DNA methylation is responsive to environment signals but changes less rapidly than mRNA or protein/metabolite levels, it may represent a more stable bio-archive of environmental exposures [20–22]. Importantly, these genome-wide chemical “annotations” hold considerable promise as molecular indicators of toxic exposures and possible predictors of disease risk [16, 17].

Our work aims to apply recently validated biostatical and biotechnological platforms to elucidate further the connection between epigenetic changes in genes with ambient air pollution exposures *in vivo*. To this end, we evaluated methylation microarray data from a subset of 141 male participants in the Normative Aging Study (NAS), together with air pollution data from their corresponding geographical regions. We focused on a key molecular pathway and its downstream target—MAPK (the mitogen-activated protein kinase) and NF-κB (nuclear factor kappa-light-chain-enhancer of activated B cells), respectively—which have been shown to be activated by PM exposure and may operate as biological mediators of pathophysiological responses to PM [23–28].

The MAPK cascade transduces a broad range of extracellular stress and physiological signals, and it mediates cellular responses to diverse processes from cell proliferation and differentiation to inflammation [29–31]. The various signaling branches of this pathway share a multi-tiered control system: MAPK proteins are activated by dual phosphorylation on tyrosine and serine/threonine residues *via* an upstream layer of dual-specificity kinases, MAPK kinases (MAPKK), which are themselves phosphorylated by a third tier of kinases, MAPKKKs. Previous experimental work has already identified activation of the MAPK cascade as a possible mechanistic link between air pollution exposures and respiratory and cardiovascular health outcomes [27, 28]. MAPK signaling typically originates from physiological stimuli, *e.g.*, *via* cell-surface receptors—and in some instances these receptors are coupled to small GTPases [29].

Although a comprehensive MAPK signaling-interaction map, or “interactome,” has recently been created [32], drawn using experimental data from molecular studies and predictive bioinformatic models, very little is known about the epigenetic regulation of these MAPK pathway genes, individually or collectively, in humans. During signaling, communication is known to exist between the MAPK network (upstream) and associated players (downstream) like NF-κB [24]. Despite some cell-based studies which have found that PM causes expression of NF-κB-related genes and oxidant-dependent activation of NF-κB *in vitro*[25], and that air pollution particles activate NF-κB on contact with airway epithelial cell surfaces in a rodent tracheal explant model [26], *in vivo* data are lacking.

Our results demonstrate, for the first time, that methylation signals in certain (epi)gene clusters of the MAPK pathway are significantly associated with ambient air pollution exposure *in vivo*. Within the MAPK pathway, DNA methylation showed responses in genes that were specific to BC and BC with sulfates. Strikingly, the combination of BC and sulfates induced DNA methylation responses that were wholly different from those detected in BC or sulfates alone. These new data, therefore, provide evidence that the biological effects of airborne particles on the human epigenome may vary depending on emission source as well as on the combination of PM components [10, 14, 15]. In our analyses, no significant associations were found with the NF-κB pathway.

## Methods

### Human cohort description

The Normative Aging Study (NAS) is a longitudinal study of human aging in Eastern Massachusetts, established in 1963 by the Veterans Administration [13, 33–36]. Community-dwelling men from the greater Boston metropolitan area were screened at entry and accepted into the study if they had no prior history of heart disease, hypertension, diabetes mellitus, cancer, peptic ulcer, gout, recurrent asthma, bronchitis, or sinusitis. Between 1963 and 1968, a total of 2,280 men were enrolled, ranging in age from 21 to 80 years (mean = 42 years) at entry. Since their enrollment, the participants have undergone comprehensive clinical examinations at 3–5 year intervals. As part of those examinations, many clinical measures have been obtained, ranging from blood pressure (systolic and diastolic) and smoking status to complete blood count (CBC) data, as described previously [37, 38]. In the NAS, DNA has been extracted from leukocytes and stored in all visits since 1999. We conducted an epigenome-wide scan of the promoter regions of ~19,000 genes on 141 subjects from the NAS. The subjects were selected based on having sufficient DNA for the assay, while leaving DNA for subsequent studies.

Importantly, in our analyses, five blood cell proportions (obtained from the CBC data) were included as covariates in our models to account for any appreciable changes in blood composition: lymphocytes, neutrophils, monocytes, basophils, and eosinophils [37, 38]. Moreover, to slightly increase our statistical power, blood cell proportions for two participants were derived by single imputation [39–41], given that Infinium HumanMethylation450 BeadChip array data (from another study [20]) were available in the absence of CBC data. Computationally, we used the minfi package function “estimateCellCounts(),” which applies the regression-calibration approach from Houseman *et al*. [39, 42], using the flow-sorted end members from Reinius *et al*. [43]. Furthermore, to estimate those cell proportions that are not part of the Houseman *et al*. study—*i.e.*, subdividing granulocytes into neutrophils, eosinophils, and basophils—we multiplied the estimated proportion of granulocytes obtained from the Houseman method by the mean proportion among NAS participants with measured proportions (*e.g.*, % neutrophils among total granulocytes).

### Chromatin immunoprecipitation (ChIP) and DNA methylation microarray

DNA samples were hybridized to the RefSeq 385K Promoter tiling array (Roche NimbleGen, Madison, WI) representing the promoter regions of all well-characterized genes in the RefSeq database (RefSeq genes with NM Prefix), in addition to all of the UCSC-annotated CpG islands. The array coverage is based on 50-75mer probes with approximately 100 bp spacing, depending on the sequence composition of the region. Sample immunoprecipitation, labeling, hybridization and data extraction were all performed according to standard procedures optimized by Roche-NimbleGen, as previously reported by Selzer *et al*. [44].

High-quality genomic DNA (~5 μg) was isolated from blood buffy coat using QiAmp DNA blood kits (QIAGEN, Hilden, Germany) and digested with 24U Mse I (5’-T^▼^TAA) enzyme (New England BioLabs) to produce small fragments of approximately 200 bp–1 kb. This fragmented DNA was heat-denatured to produce single-stranded DNA, then immunoprecipitated using an anti-5mC (Abcam-ab10805) monoclonal mouse antibody. Methylated DNA immunoprecipitated (MeDIP) fragments were then heat-denatured for 10 min at 95°C and immediately cooled on ice. Immune complexes were captured with Protein-A agarose bead slurry (Invitrogen-15918-014) and washed to remove non-specifically-bounded material. Following elution of bound complexes, MeDIP samples were purified with phenol-chloroform:isoamyl alcohol and ethanol precipitation in a −80°C freezer for 30 min. After centrifugation, the supernatant was carefully removed, and the pellet was washed with cold 70% ethanol and then centrifuged again to remove residual supernatant. MeDIP samples were completely air dried and re-suspended in 30 μl of 10 mM Tris HCl (pH 8.5). Fragments were amplified by whole-genome amplification (GenomePlex® Complete Whole Genome Amplification [WGA2] Kit, Sigma-Aldrich). Experimental and total DNA samples were labeled using 9mer primers, with Cy3 and Cy5 dyes attached *via* Klenow labelling (50 units/μL, New England BioLabs). The labeled experimental IP and total DNAs were co-hybridized to the array for 16–20 hours, washed, and scanned by the Roche NimbleGen Service Laboratory (Reykjavík, Iceland)*.* The intensity ratio of IP to total DNA was used to identify DNA methylation.

### Ambient air pollution modeling

Continuous air pollution concentrations were measured at a Harvard School of Public Health monitoring site located on the Francis A. Countway Library of Medicine rooftop (10 Shattuck Street, Boston, MA), 1 km from the clinical examination site. Average pollution measures for the month prior to the blood draw were computed. BC, a marker for traffic particles weighted toward diesel particles, was measured using an aethalometer (Magee Scientific, Berkeley, CA), and PM_2.5_ was measured using a Tapered Element Oscillating Microbalance (model 1400A; Rupprecht & Pataschnick Co., East Greenbush, NY), operated at 50 degrees with two 4 liter per minute PM_2.5_ impactors before the inlet. From September 25, 1999 to February 2, 2004, particulate sulfate was measured using the Harvard/EPA Denuder System (HEADS), which samples inorganic gaseous and particulate species in the air. From January 1, 2003 through 2007, daily particulate filter samples were analyzed, by X-ray fluorescence (XRF) spectroscopy, for elemental components. From these samples, we multiplied the mass of sulfur by three to obtain the mass of sulfate. For the days when both HEADS impactors and XRF were in operation, we used linear regression and determined that the measurements had a slope of 1 and R^2^ > 0.9, indicating a high correlation between the two monitoring methods. XRF measurements were used during this period of overlap. These sulfate particles are secondary, long-range particles primarily from coal-burning power plants.

### Normalization and pre-processing of DNA methylation data

We normalized the raw methylation intensities (log2 green *vs*. red channel ratio) for each probe by subtracting the overall median and then dividing by the probe’s GC-content specific standard deviation, which is the standard deviation of all the probes whose sequence has the same number of G and C nucleotides as the target probe. We then smoothed the normalized scores using a local linear kernel smoother, as in Fan and Gijbels (1996), over the probe locations. For a given gene, its methylation score was then calculated by taking the area under the smoothed curve, truncated at zero, over a 500 bp window around the transcription start site of the gene, and then dividing by the percentage of CpG dinucleotides in the DNA sequence within the window and the number of probes having positive scores within the window.

### Biostatistical methods and pathway analysis

We identified the genes associated with the MAP kinase and NF-κB signaling pathways using the BioCarta reference website. Since the outcome (gene methylation) is high dimensional, we employed a canonical-correlation-analysis (CCA)-based approach, which is a type of extension of the usual regression model for multiple outcomes. Under this approach, to adjust for confounders, we first regressed each of the exposures of interest, and the outcomes of interest, on the set of confounders, and then used the residuals from these regressions in the analysis.

For each gene pathway, we performed three analyses. We studied the association of the gene methylations in the pathway with (i.) BC exposure alone, adjusting for age, blood pressure, smoking status, blood cell composition, and sulfate exposure as confounders, (ii.) the association of gene methylation with sulfate alone, adjusting for the aforementioned confounders and BC exposure, and (iii.) joint association modeling in which we studied the association of the pathway with both BC and sulfate exposure jointly (while adjusting for all other confounders). Our goal was to identify exposure-specific effects, but also to identify the effect of air pollution in the more realistic scenario in which people are exposed to *both* pollutants.

As mentioned above, we used a sparse forward stepwise-CCA method to identify specific genes that contribute to the association between the exposure and the DNA methylation status in the gene pathway. In short, the exposure set is held fixed, while at each step, the gene methylation score that contributes most to the association (between the gene set and the exposure) is selected. Genes are added to the set until a score is maximized. The association between the exposure and methylations is measured using the canonical correlation between the two sets, namely **X** and **Y**. The canonical correlation is given by cor(**Xa**, **Yb**), where **a** and **b** are weight vectors (also called loading vectors), with lengths representing the number of measures in the exposures (pollutants) as “set **X**,” and the outcomes (gene methylations) as “set **Y**,” which are calculated to maximize the canonical correlation under the constrain **a**^T^cov(**X**)**a** =1**b**^T^cov(**Y**)**b** =1. Each entry in **a** is a weight corresponding to a specific pollutant (exposure), and each entry in **b** is a weight corresponding to a specific gene methylation measure. The larger the weight (in absolute value), the larger the influence of the variable it represents on the canonical correlation.

The score used as a criterion to select genes was the empirical CIC (Correlation Information Criterion), which takes the correlation between the identified set of genes and the exposures and removes the 99^th^ percentile of this distribution under the null. This distribution was determined by 1000 random samples for each combination of 1 or 2 exposures (depending on the particular exposure model of interest) and any number of “outcomes” (1, 2, 3, 4, .…, number of genes in the pathway under study).

To test the significance of the canonical correlation between the set selected (set of “outcomes” and the set of exposures) we used the Wilks’ Lambda tests statistic, applied with a permutation procedure. The Wilks’ Lambda given by the ratio det(cov(**X**,**Y**))/[det(cov(**X**))det(cov(**Y**))], where det(**C**) is the determinant of a matrix **C**, and (**X**,**Y**) is the data matrix of both the exposures **X** and the outcomes **Y**, is used to test the null hypothesis of no association between two data matrices **X** and **Y**. It cannot be used on the selected data because the variable selection method (stepwise-CCA) was applied, changing the null distribution of the correlation between the outcomes and the exposures. Thus we permuted the exposure data while holding the methylation scores for all genes in the pathway, fixed 3000 times, then performed the stepwise analysis on the permuted data, and finally computed the Wilks’ Lambda statistic for the exposure and genes identified by the stepwise-CCA method. The P-value for the true exposure is the proportion of permuted exposures with a lower Wilks’ Lambda P-value than the one for the true exposure.

### Bioinformatic visualization of an integrated MAPK network

Pathways from the hiPathDb [45] Integrated database were selected to link all BioCarta MAPK genes; these pathways included the KEGG [46, 47] ErbB signaling and MAPK signaling pathways (path:hsa04010 and path:hsa0401, respectively), in addition to the BioCarta MAPK signaling pathway (pid p 100113 mapkpathway). The integrated pathways were downloaded in XML format and imported into Cytoscape [48]. All non-gene nodes were removed by hand, and the network was restricted to second neighbors of the BioCarta MAP kinase genes. Supernodes with only a single connection or only single direction edges were removed, and redundant supernode-to-supernode connections were simplified whenever possible; self-directed loops and redundant edges of the same direction were also removed. Nodes were annotated by fill-color with methylation coefficients on a truncated scale of −1.5 to 1.5, as shown in the legend.

### Disease ontologies of selected genes

The MAPK (epi)gene hits were analyzed for disease association without regard to enrichment with the GeneAnswers library [49]. Selected genes (11 total) comprised those with non-zero methylation coefficients for any of the three exposure conditions (black carbon, sulfate, and multi-pollutant carbon and sulfate). GeneAnswers associates genes with disease using DOLite [50], a database based on the Disease Ontology [51], an open-source ontology for the semantic integration of biomedical data associated with human disease.

## Results and discussion

### Novel (epi)gene pathway-exposure assessment

We hypothesized that short-term exposure to environmental air pollution components, singly or in a multi-pollution paradigm, would be associated with blood DNA methylation alterations in known inflammation-linked gene networks, specifically the MAPK pathway, and possibly in a downstream target, NF-κB. To study this hypothesis, we implemented a multidisciplinary strategy with an established sparse stepwise canonical correlation analysis (stepwise-CCA) method [52, 53], by which we were able to evaluate genes in the abovementioned pathways with respect to air pollution-related epigenetic alterations.

We first performed epigenome-wide scans of promoter regions for ~19,000 genes from 141 participants from the Normative Aging Study [37, 54, 55]. Briefly, our previously validated workflow (described in Methods) included DNA fragmentation, methylated DNA immunoprecipitation (MeDIP) capture, DNA purification, hybridization to the RefSeq 385K Promoter tiling array for methylomic detection, and (epi)gene clustering by methylation status *via* the stepwise-CCA algorithm [53]. Using the BioCarta reference database [56], we identified methylation changes in the promoters of 84 genes from the MAP kinase-signaling pathway, which are listed in Table 1. In humans, this large gene network has very rarely been evaluated within the context of air pollution-related effects *in vivo*.

**Table 1:** **The 84 MAPK pathway-linked genes considered in our analyses**

	Gene		Gene		Gene
01	*ATF2*	38	*MAP3K5*	73	*TRADD*
02	*CREB1*	39	*MAP3K6*	74	*TGFB1*
03	*CEBPA*	40	*MAP3K7*	75	*TGFB2*
04	*CHUK*	41	*MAP3K8*	76	*TGFB3*
05	*DAXX*	42	*MAP3K9*	77	*TGFBR1*
06	*ELK1*	43	*MAP4K1*	78	*FOS*
07	*GRB2*	44	*MAP4K2*	79	*HRAS*
08	*IKBKB*	45	*MAP4K3*	80	*MYC*
09	*JUN*	46	*MAP4K4*	81	*ARAF*
10	*MKNK1*	47	*MAP4K5*	82	*BRAF*
11	*MKNK2*	48	*MAPKAPK2*	83	*RAF1*
12	*MAPK1*	49	*MAPKAPK3*	84	*RELA*
13	*MAPK10*	45	*MAP4K3*		
14	*MAPK11*	50	*MAPKAPK5*		
15	*MAPK12*	51	*MAX*		
16	*MAPK13*	52	*MEF2A*		
17	*MAPK14*	53	*MEF2B*		
18	*MAPK3*	54	*MEF2C*		
19	*MAPK4*	50	*MAPKAPK5*		
20	*MAPK6*	55	*MEF2D*		
21	*MAPK7*	56	*NFKB1*		
22	*MAPK9*	57	*NFKBIA*		
23	*MAP2K1*	58	*PAK2*		
24	*MAP2K2*	59	*PAK1*		
25	*MAP2K3*	60	*RAC1*		
26	*MAP2K4*	61	*RIPK1*		
27	*MAP2K5*	62	*RPS6KB1*		
28	*MAP2K6*	63	*RPS6KB2*		
29	*MAP2K7*	64	*RPS6KA1*		
30	*MAP3K10*	65	*RPS6KA2*		
31	*MAP3K11*	66	*RPS6KA3*		
32	*MAP3K12*	67	*RPS6KA4*		
33	*MAP3K13*	68	*RPS6KA5*		
34	*MAP3K14*	69	*SHC1*		
35	*MAP3K2*	70	*STAT1*		
36	*MAP3K3*	71	*SP1*		
37	*MAP3K4*	72	*TRAF2*		

To our knowledge, this is the first human study to systematically evaluate MAPK gene-promoter methylation using a wide collection of upstream and downstream pathway components with respect to ambient air pollution exposures.

We normalized the raw methylation intensities for each array probe separately, smoothed these normalized intensities for each probe, and finally constructed gene-specific methylation scores based on the intensities of neighboring probes around the transcription start site (TSS) of each gene, as previously described [53]. We used these gene-specific methylation scores, or “weights,” to investigate the association between DNA methylation and environmental exposures.

The gene methylation weights found by the stepwise-CAA algorithm are provided throughout all of the data tables: A large weight in the absolute value is interpreted as a stronger effect. A positive value indicates increased methylation (hypermethylation) with increased exposure to BC (or sulfate, *etc*.), and a negative weight implies decreased methylation (hypomethylation) with increased exposure. These weighted coefficients, however, should not be interpreted individually—but rather as a combined (epi)gene cluster “hit,” constituting a set of bi-directional epigenetic marks (+ or -) among pathway genes associated with an exposure model.

### Human cohort characteristics

Table 2 shows a summary of the characteristics of the subgroup of NAS participants from our methylation study for which sulfate measures were available (n = 90) *vs.* the larger set. The characteristics reported for this subgroup—BC, age, and other measures—were almost identical to those calculated using information from the entire participant list (n =141). The air pollution measures are averages of the 30 days of ambient BC and/or sulfate concentration prior to the day that blood was collected for methylomic analysis. This time window was selected to balance the evidence of effects of short-term air pollution exposure on the cardiovascular and respiratory systems, with the common understanding that at least some potential environmental effects on DNA methylation require days (or weeks) to become apparent [57–59].

**Table 2:** **Summary of relevant NAS characteristics used in this study: complete set and subset of participants who had sulfate measures available (n =90), out of a total of 141 participants**

NAS cohort description	All participants	With sulfate measures
**Age (years)**	n = 141 people	n = 90 people
	Median 73; Range 56–88; SD ±6.8	Median 73; Range 58–88; SD ±6.6
**Blood pressure (mmHg)**	n = 141	n = 90
Systolic	Median 130; Range 87–188; SD ±16.13	Median 129.5; Range 87–188; SD ±15.65
Diastolic	Median 79; Range 54–98; SD ±9.1	Median 78; Range 55–96; SD ±9.11
**Exposure (μg/m^3)**	n = 141	n = 90
BC, 30-days averaged	Mean 0.84; SD ±0.16	Mean 0.83; SD ±0.15
Sulfate, 30-days averaged	N/A	Mean 3.06; SD ±0.79
**Smoking status (n)**	n = 141	n = 90
Never	53	32
Ever	7	5
Former	81	53
**Blood count (%)**	n = 136	n = 88
Lymphocytes	Median 26; Range 6–39; SD ±6.66	Median 26; Range 6–38; SD ±6.92
Neutrophils	Median 62; Range 45–86; SD ±7.71	Median 63; Range 45–86; SD ±7.78
Monocytes	Median 9; Range 4–14; SD ±1.92	Median 8; Range 4–14; SD ±1.96
Basophils	Median 1; Range 0–2; SD ±0.51	Median 1; Range 0–2; SD ±0.52
Eosinophils	Median 3; Range 0–11; SD ±1.9	Median 3; Range 0–10; SD ±1.83

Relevant units are supplied in the left-hand side for each characteristic, with median (or mean), range, standard deviation, and sample size indicated.

Our data included 84 MAPK pathway genes (listed in Table 1), from which we identified exposure-specific (epi)gene sets (Table 3) based on three pollution models. We identified 11 genes whose methylation status was associated with BC exposure (P-value 0.04) after adjusting for relevant confounders: age, sulfate exposure, blood-cell-type proportions (derived from the CBC data), smoking status, and blood pressure (see Methods). Association analysis between sulfate exposure and DNA methylation in the MAPK pathway identified 12 genes after adjusting for BC exposure and the other aforementioned confounders, yet this group fell below the level of statistical significance (P-value 0.10) likely due to a smaller sample size (n = 90). Finally, multi-pollutant analysis of DNA methylation associated jointly with sulfate and BC exposure yielded 14 significant genes (P-value 0.01).

**Table 3:** **Results of the stepwise-CCA algorithm applied to the MAP kinase pathway genes, grouped by exposure-specific model**

	Weighted coefficients by exposure model		
ID	Black Carbon (BC)	Sulfates (S)	Multi (BCS)
BC	1	0	0.49
S	0	1	−1.08
*MKNK2*	0.39	0	0
*MAPK10*	0	0.5	−0.36
*MAPK13*	0.29	−0.51	0
*MAPK6*	0.65	0	0
*MAPK9*	0	0.13	−0.22
*MAP2K1*	0	0	−0.3
*MAP2K5*	0	0	0.42
*MAP2K6*	0	0	0.22
*MAP3K11*	0	−0.4	0
*MAP3K14*	0	0	0.37
*MAP3K2*	−0.25	0	0
*MAP3K6*	−0.37	0	0
*MAP3K7*	0	0.41	0
*MAP4K1*	0	−0.19	0.31
*MAP4K3*	0.42	0	0
*MAP4K4*	0	0.57	−0.61
*MAPKAPK2*	−1.36	0.62	0
*MEF2A*	0.32	0	0.33
*PAK1*	0	0	−0.57
*RPS6KB1*	0	−0.28	0.09
*RPS6KB2*	0.27	0	0
*RPS6KA3*	0	−0.33	0
*SHC1*	0.33	0	0
*STAT1*	0	−0.49	0.43
*TGFB1*	0	0.28	0
*MYC*	0	0	0.23
*RELA*	0.27	0	0.48
Canonical correlation	0.73	0.73	0.78
P-value*	0.02	0.04	0.05
P-value**	0.04	0.10	0.01

We identified a cluster of 27 MAPK gene hits, and their corresponding weights (as coefficients after 3K permutation tests) are shown. At the bottom of the table, P-values for each model are labeled as either adjusted for all covariates, with two asterisks (**), or for all covariates except blood cell proportions, with one asterisk (*).

Importantly, adjusting for blood cell proportions barely influenced the effect estimates, so in Table 3, we report both P-values, with and without the cell proportion adjustment (while including all of the other confounders). In Additional file 1, A-D, we present clustered heatmaps of the correlations between the full list of 84 MAPK pathway genes (from Table 1) *vs*. the cluster of 27 gene hits (Table 3), before and after adjusting for all confounders.

**Figure 1:**
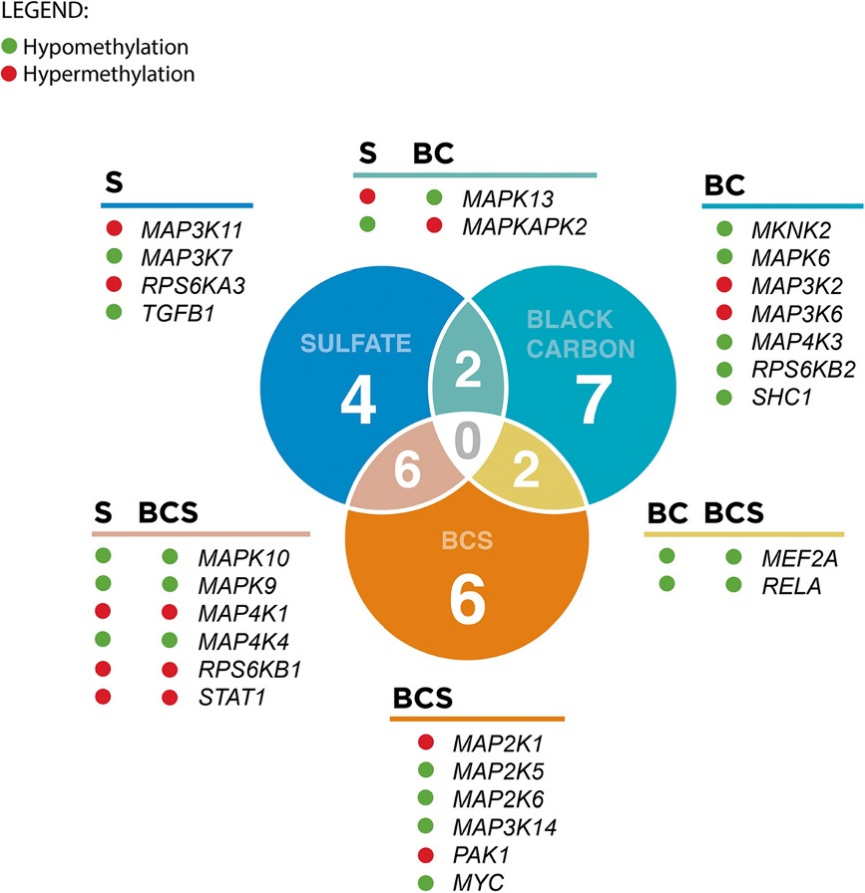
**Summary of all possible relationships between MAPK pathway gene hits as grouped by exposure model.** All of the MAPK genes from Table 3 are grouped here by their exposure-specific model: black carbon (BC); sulfates (S); and multi-exposure for BC and sulfates (BCS). The various sections of this Venn diagram are color-coded to help identify gene subgroups within each region of the figure. The DNA methylation status of each gene is summarized as either increased (green) or decreased (red).

To aid best in visualizing all possible relationships amongst our various MAPK hits from Table 3, we next constructed an annotated Venn diagram (Figure 1). Interestingly, no genes occupied the union of all 3 exposure models, and minor overlap was observed across any 2 given exposure combinations. For example, only two genes—*MAPK13* and *MAPKAPK2*—overlapped between BC and sulfate, yet their methylation status reversed directionality. Strikingly, it is evident that the genes associated with the multi-pollutant model (BCS) are not simply the aggregate collection of hits found in the BC and sulfate models. In fact, it behaves like an entirely novel exposure combination, suggesting that multi-pollutant exposures may impact the epigenome in disparate ways, unlike their single-exposure counterparts.

This key observation is also consistent with our past work to identify new methylomic changes in gene promoters related to the asthma pathway: Only a single allergy-specific receptor gene, *FCER1G*, exhibited methylation changes associated with both BC and sulfate exposure [53]. All other significant immune-gene clusters were either specific to BC exposure alone (6 genes) or to sulfate exposure (4 genes). Functionally, however, all of these genes did share a common biological network across immune cells and the bronchus, which could be easily visualized.

### Visualization of MAPK pathway components

**Figure 2:**
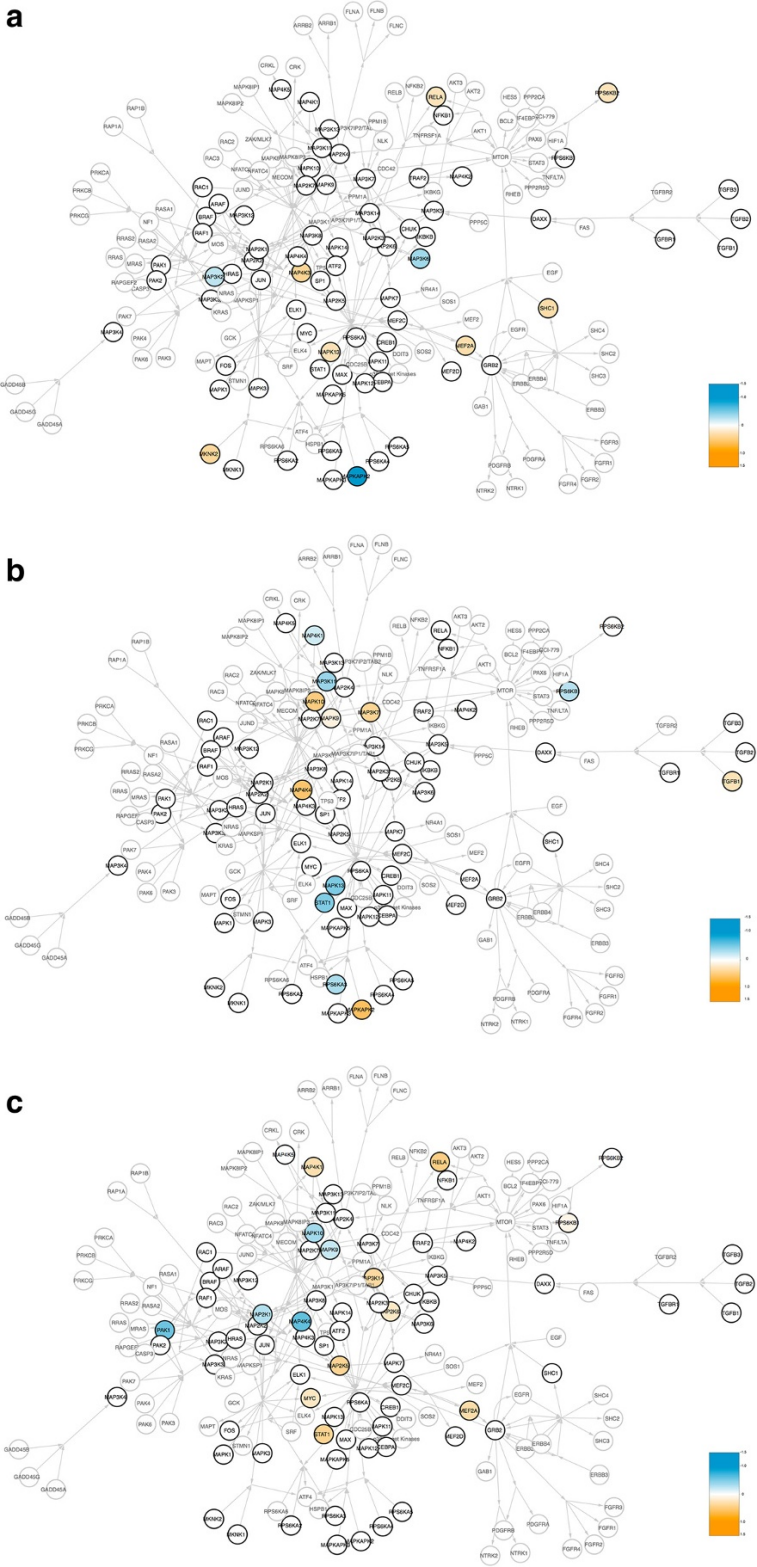
**Methylation coefficients of our (epi)gene hits within the broader MAPK signaling system.** A nexus integrating all BioCarta MAP kinase genes to other previously studied MAPK networks is diagramed, wherein nodes representing the genes within the BioCarta MAP kinase pathway (84 total) are outlined and labeled in dark black. Arrows indicate known direction of action. Methylation coefficients (from Table 3) are represented here in a scale from blue (negative values), to white (zero), to orange (positive values). For simplicity, both unmeasured values and zero are represented in white. Exposure-specific MAPK coefficients are shown across all three of our models: **(a)** black carbon; **(b)** sulfates; and **(c)** the multi-pollutant paradigm.

In the present work, however, our 27 (epi)gene hits in the MAPK pathway do not share any obvious physiological link, collectively, within some unified cardiopulmonary pathway relevant to air pollution—thus perhaps constituting a novel epigenetic/signaling “crossroads” of exposure-associated genes relevant to the processing of environmental PM signals *in vivo*. To help visualize this dynamic system, we next used an integrated bioinformatic approach to overlay our DNA methylation coefficients (from Table 3) onto an expanded MAPK signaling map. In Figure 2, we can best appreciate the following points: (i.) our initial set of 84 MAPK genes (dark circles) map widely across the broadened MAPK signaling system, thereby ensuring that most branches of the network were aptly queried *via* our stepwise-CAA method; (ii.) the distribution of exposure-specific MAPK hits across the multi-pollution model (Figure 2c) is not simply an aggregate of signaling components found in the two other models, BC (2a) and sulfates (2b).

**Figure 3:**
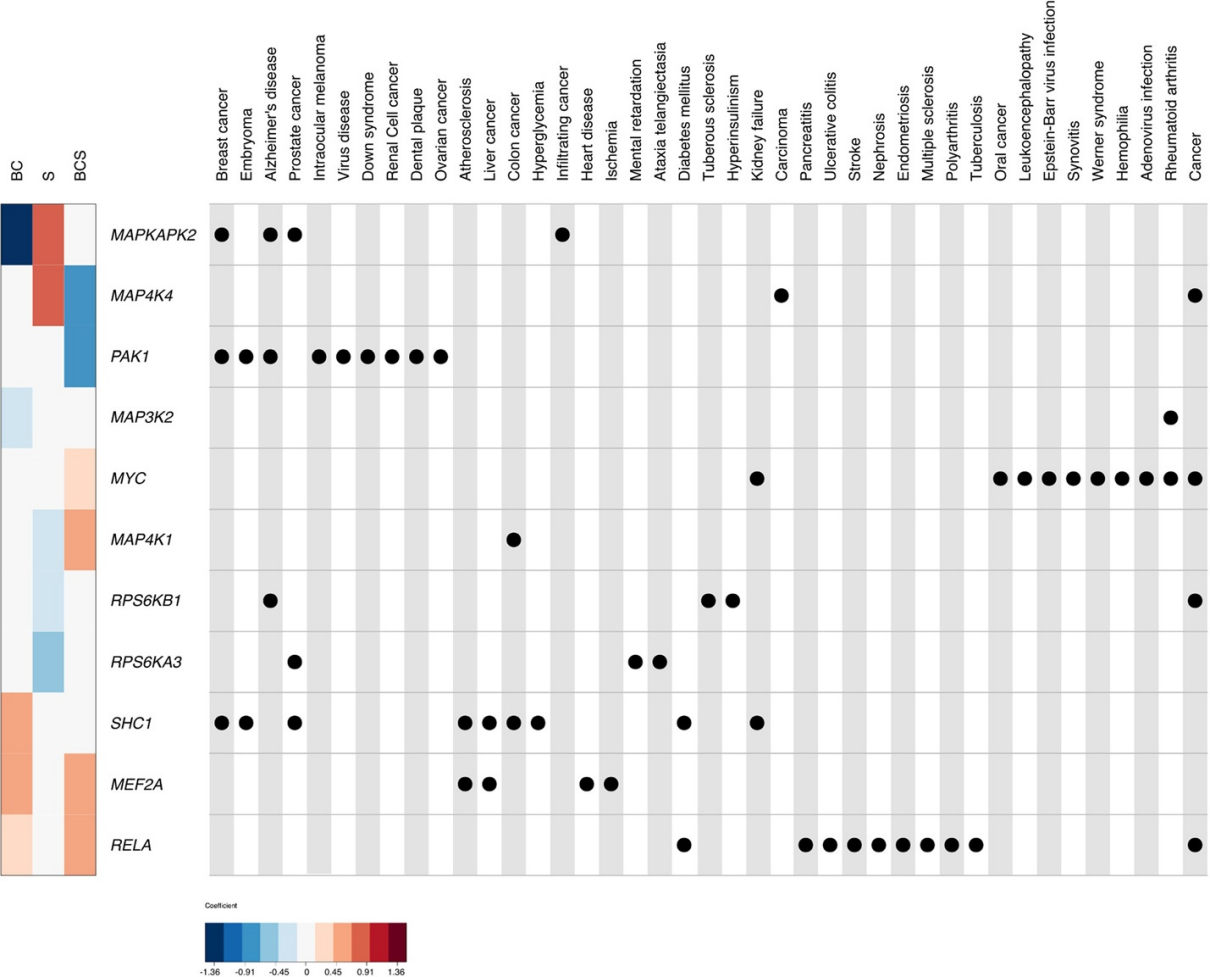
**Biocomputational profiling of disease-linked MAPK (epi)gene hits.** A heatmap (left) of methylation coefficients for the three pollution paradigms—black carbon (BC), sulfates (S), and BC with sulfate (BCS)—and a corresponding disease ontology table (right) are shown. Only genes with an annotation within the disease ontology [51] are shown: *i.e*., 11 out of 27 MAPK genes (~41%). Heatmap colors represent the methylation coefficients, with negative values in blue, zero in white, and positive values in red. Rows within both the heatmap and the concept-map represent individual genes as noted. Columns within the disease ontology concept-map represent individual diseases within the disease ontology. Disease category hierarchies were collapsed, so diseases may represent subcategories of other diseases represented (*e.g*., “Breast cancer” and “Ovarian cancer” are both sub-categories of “Cancer”). Dots within the disease ontology table denote an association of a gene with a given pathology. Alternative shading is used to help demarcate columns.

In light of these data, therefore, we next hypothesized that perhaps some of these genes (across all 3 exposure models) would be linked to relevant human disease outcomes already known to be exacerbated/modulated by air pollution—*e.g.*, heart disease [60, 61], atherosclerosis [62–64], stroke [65, 66], cancers [67–69], *etc*.—among various other disease contexts. Indeed, biocomputational profiling (Figure 3) confirmed that 11 of our 27 hits (~41%) were in fact highly associated with a wealth of PM-linked pathophysiological conditions (and to many other varied diseases), thereby underscoring that these genes may serve as an epigenetic/signaling nexus of exposure-related signals *in vivo*, a role not fully appreciated among their other biological functions.

Importantly, some individual connections between our MAPK gene hits and various air pollution exposure models do exist, upon examining a handful of *in vitro* cell-based and/or animal-exposure studies. This literature helps to further confirm the biological relevance of the MAPK pathway within air pollution models and paradigms.

For example, the MAP2K1 protein (also known as MEK1, MAPKK1, or MKK1) was previously found to be important for induction of COX-2 protein expression *via* vanadate exposure *in vitro* using a human lung carcinoma cell line (A549) [70]. Moreover, another group reported that primary human pulmonary cells exposed to cigarette smoke extract (CSE), especially normal human bronchial smooth muscle cells, required MAPKAPK2/MK2 protein activation to promote pathological expression of pro-inflammatory Interlukin-8 [71]. Lastly, two key downstream transcription factors associated with MAPK signaling merit special consideration, STAT-1 and MYC. Vehicular-derived airborne nano-sized particulate matter (from Los Angeles, CA) that was re-aerosolized and administered to C57Bl/6 J male mice led to increased MYC expression in brain (cerebellum), liver, and lung tissue [72], and vanadium compounds present in PM, such as vanadium pentoxide, induced STAT-1 activation in lung myofibroblasts, which required upstream hydrogen peroxide and MAP kinase signaling activity [73].

In our study, the associations between the abovementioned MAPK pathway genes and our exposure models were so specific (and reproducible) that further attempts to find other significant (epi)gene clusters with the NF-κB pathway, a downstream network often coupled to MAPK signaling, failed consistently, despite a ~36% overlap in gene sets across both pathways, as noted in Tables 4 and 5. We do recognize, however, that perhaps a higher sample size could have helped to facilitate an association, which may not have been possible given our current number of participants. Moreover, we also acknowledge other key limitations inherent to our work, given that the NAS is a male-only cohort and that we analyzed blood-cell DNA.

**Table 4:** **Complete list of the NF-κB signaling pathway and associated genes (22 total) considered in this study**

	Gene		Gene
01	*CHUK* ^†^	12	*NFKB1* ^†^
02	*FADD*	13	*NFKBIA* ^†^
03	*IKBKB*	14	*RIPK1* ^†^
04	*IKBKG*	15	*TRAF6*
05	*IL1R1*	16	*TRADD* ^†^
06	*IL1A*	17	*TLR4*
07	*IRAK1*	18	*TNF*
08	*MAP3K14* ^†^	19	*TNFRSF1A*
09	*MAP3K7* ^†^	20	*TNFRSF1B*
10	*TAB1*	21	*TNFAIP3*
11	*MYD88*	22	*RELA* ^†^

Despite the fact that 8 out of these 22 genes denoted by a dagger (†) were shared by the MAPK pathway list in Table 1 (~36% overlap), our stepwise-CCA method failed to identify any statistically significant NF-κB gene clusters for any of the three exposure paradigms.

**Table 5:** **No significant associations were obtained between NF-κB genes and the exposure models tested**

	Weighted coefficients by exposure model		
ID	Black Carbon (BC)	Sulfates (S)	Multi (BCS)
BC	1	0	−0.49
S	0	1	1.08
*MYD88*	−0.96	0	0
*RELA*	0	−0.54	0
*TNFRSF1A*	0	0.82	1
*TNFRSF1B*	1.15	0	0
Canonical correlation	0.23	0.28	0.24
P-value*	0.88	0.61	0.81
P-value**	0.98	0.54	0.84

P-values for each model are indicated as either adjusted for all covariates (**) or all covariates except blood cell proportions (*).

### Limitations and relevant considerations

Since the NAS is a male-only cohort, comprised of aging individuals, we caution that any of the significant (epi)gene-exposure associations observed here may not hold exactly the same in females. Broadly speaking, the observation that DNA methylation in the promoters of MAPK genes is altered by common ambient exposures (either in a single- or multi-pollutant paradigm) is unlikely to be a response inherent only to elderly men—yet, both the magnitude of the response and which specific gene candidates are most associated with the exposure may certainly be influenced by age/sex [74, 75].

This study utilized peripheral leukocyte DNA for methylomic evaluation, as we have previously published using similar methodologies [53]. Since lung and/or cardiac tissue is impractical (and complex) to obtain from healthy participants, most human *in vivo* exposure investigations rely on blood-based discovery platforms. Circulating leukocytes activated by PM exposure have been suggested to mediate and/or amplify, through immune and inflammatory pathways, the effects of air pollution on the cardiovascular and respiratory systems [76–78]. Importantly, our conceptual framework does not assume that the blood methylome is necessarily correlated with that of the heart and/or lungs.

Although we were cautious to ensure that any exposure-associated alterations in DNA methylation were not merely due to changes in the ratios of blood cells—by including blood cell proportions as key covariates in all of our models (explained in Methods)—we lack the ability to link these epigenetic marks to any appreciable modulation in gene expression in these blood cells. We acknowledge this experimental limitation given that most of our work was *in silico*. As mentioned earlier, however, other studies from colleagues—spanning a diverse spectrum of cell-culture/animal models—have demonstrated that exposure to PM and/or its components can affect expression of MAPK pathway genes (as well as MAPK protein activation) to promote cellular signaling. Remarkably, in this study with human blood cells, we identified some of the same MAPK pathway genes. Furthermore, we have also shown that some of our gene hits from blood were previously linked to disease outcomes known to be exacerbated by PM in people (Figure 3).

In light of these points, it is possible that blood-cell DNA methylation is not simply a passive, irrelevant target of airborne environmental exposures: Blood-cell methylomic alterations may eventually contribute directly/indirectly to cardiopulmonary outcomes *via* mechanisms not yet well understood, *e.g.*, *via* aberrant MAPK signaling as a consequence of (epi)gene destabilization. Bone marrow is a highly vascular tissue, so blood-borne toxicants in PM may continue to expose hematopoietic stem cells in the marrow, thereby promoting a positive feedback loop that establishes persistent methylomic alterations in the blood. To this end, the specific 27 MAPK genes identified here, whose promoter regions can undergo directional epigenetic modification (either hypo- or hyper-methylation) in response to various exposure paradigms, merit future analysis.

## Conclusions

This is the first human epigenetic study to evaluate MAPK gene-promoter methylation changes, linking alterations in 27 MAPK genes to ambient air pollution exposures *in vivo*. Although the MAPK pathway was significantly associated with two out of three exposure models tested, these models were associated with the pathway quite differently. An integrated, systems-level approach, therefore, is needed to dissect more finely single- *vs*. multi-pollutant exposure effects *in vivo.* Indeed, by further identifying and studying epigenetic changes relevant to toxic exposures, our research may provide new tools to develop targeted prevention when it is most effective, *i.e.* in early stages or, among exposed individuals, even before any subclinical cardiopulmonary impairment is detectable. As methods for accurate epigenomic profiling become increasingly available and affordable, these approaches may allow for better multi-pollutant exposure assessment to be brought to numerous environmental health studies, as well as to preventive settings where exposure data are lacking or where funds and opportunities for expensive personal monitoring are limited. As the age of the US population increases, such efforts will have the potential to help millions of individuals in the prevention of air pollution-related pathophysiological outcomes and their sequelae, particularly among vulnerable people, and to narrow health disparities and promote equity.

## Electronic supplementary material

Additional file 1: **Clustered heatmaps of the observed correlations between our full MAPK gene set and those shown to be associated with air pollution.** **(A)** Unadjusted DNA methylation coefficients were used to cluster the 84 genes listed in Table 1; **(B)** clustering of adjusted methylation coefficients, after accounting for all relevant confounders included in this study—age, blood pressure, smoking status, blood cell proportions, *etc*. (described in Methods); similarly, the same is shown for the 27 MAPK gene hits from Table 3, prior to adjusting for all confounders **(C)** and afterwards **(D)**. (ZIP 3 MB)

## Abbreviations

PM: Particulate matter
BC: Black carbon
S: Sulfate
BCS: Black carbon and sulfate
CBC: Complete blood count
MAPK: Mitogen-activated protein kinase
stepwise-CCA: Stepwise-canonical correlation analysis
NF-κB: Nuclear factor kappa-light-chain-enhancer of activated B cells
5mC: 5-methyl-cytosine
NAS: Normative Aging Study.
