# Supplementary figures and images for: Short-term airborne particulate matter exposure alters the epigenetic landscape of human genes associated with the mitogen-activated protein kinase network: a cross-sectional study
Source: Environ Health. 2014 Nov 13;13:94. doi: 10.1186/1476-069X-13-94 (PMC4273424; doi:10.1186/1476-069X-13-94)

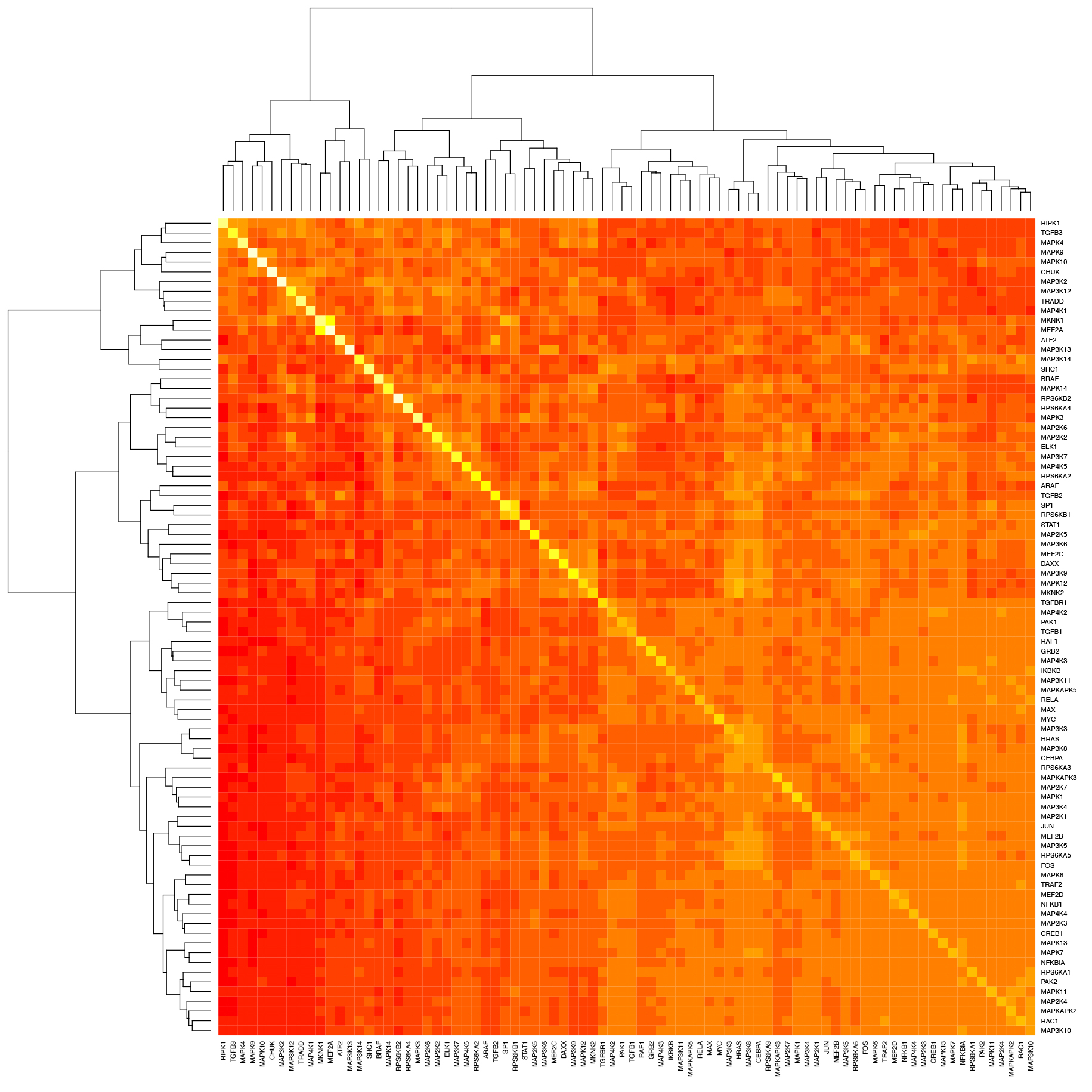

Supplement: Supplementary file 1 — Additional file 1: Clustered heatmaps of the observed correlations between our full MAPK gene set and those shown to be associated with air pollution. (A) Unadjusted DNA methylation coefficients were used to cluster the 84 genes listed in Table 1; (B) clustering of adjusted methylation coefficients, after accounting for all relevant confounders included in this study—age, blood pressure, smoking status, blood cell proportions, etc. (described in Methods); similarly, the same is shown for the 27 MAPK gene hits from Table 3, prior to adjusting for all confounders (C) and afterwards (D). (ZIP 3 MB) [file 12940_2014_810_MOESM1_ESM.zip › 10018_7989112011285506_add1/10018_7989112011285506_add1A.jpeg]

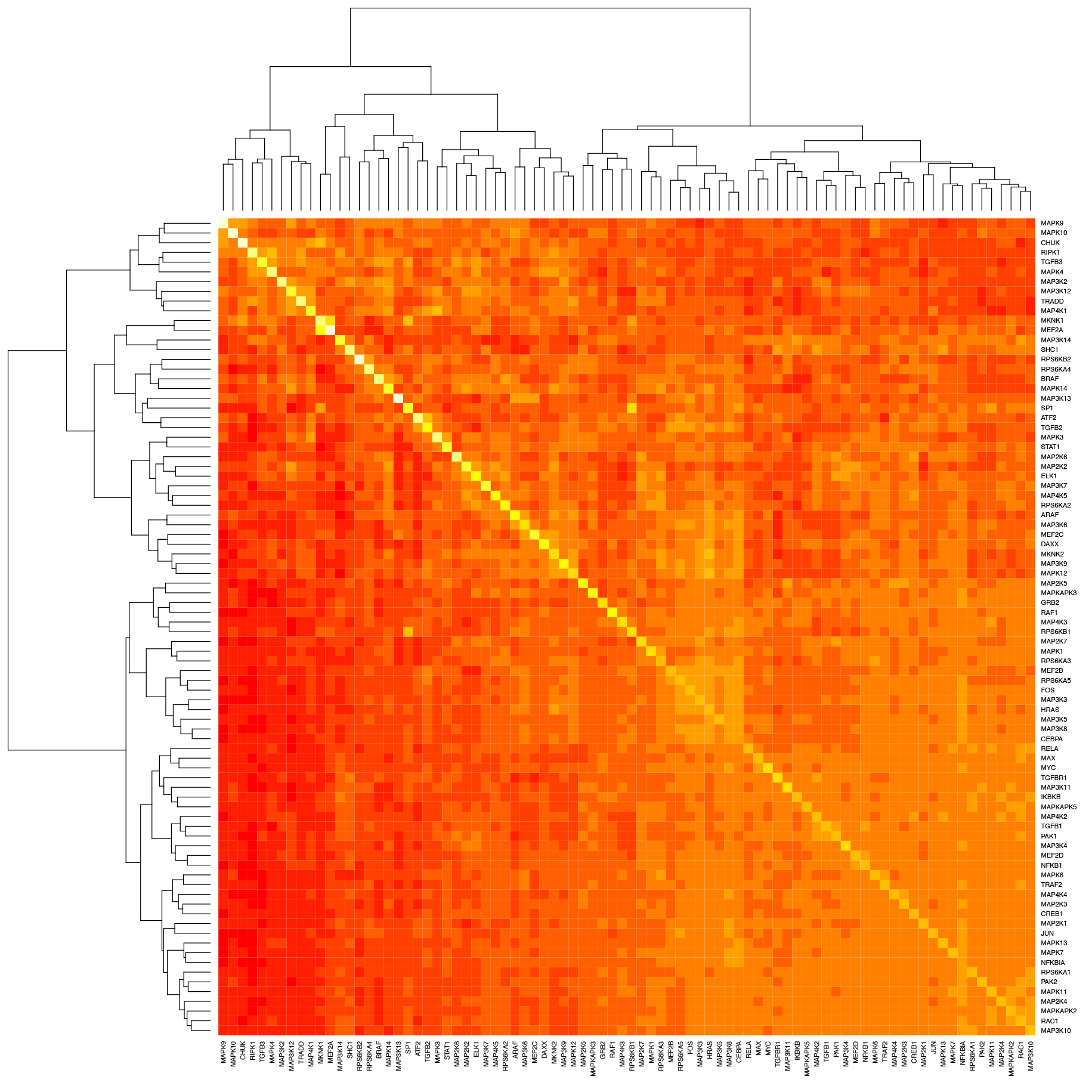

Supplement: Supplementary file 1 — Additional file 1: Clustered heatmaps of the observed correlations between our full MAPK gene set and those shown to be associated with air pollution. (A) Unadjusted DNA methylation coefficients were used to cluster the 84 genes listed in Table 1; (B) clustering of adjusted methylation coefficients, after accounting for all relevant confounders included in this study—age, blood pressure, smoking status, blood cell proportions, etc. (described in Methods); similarly, the same is shown for the 27 MAPK gene hits from Table 3, prior to adjusting for all confounders (C) and afterwards (D). (ZIP 3 MB) [file 12940_2014_810_MOESM1_ESM.zip › 10018_7989112011285506_add1/10018_7989112011285506_add1B.jpeg]

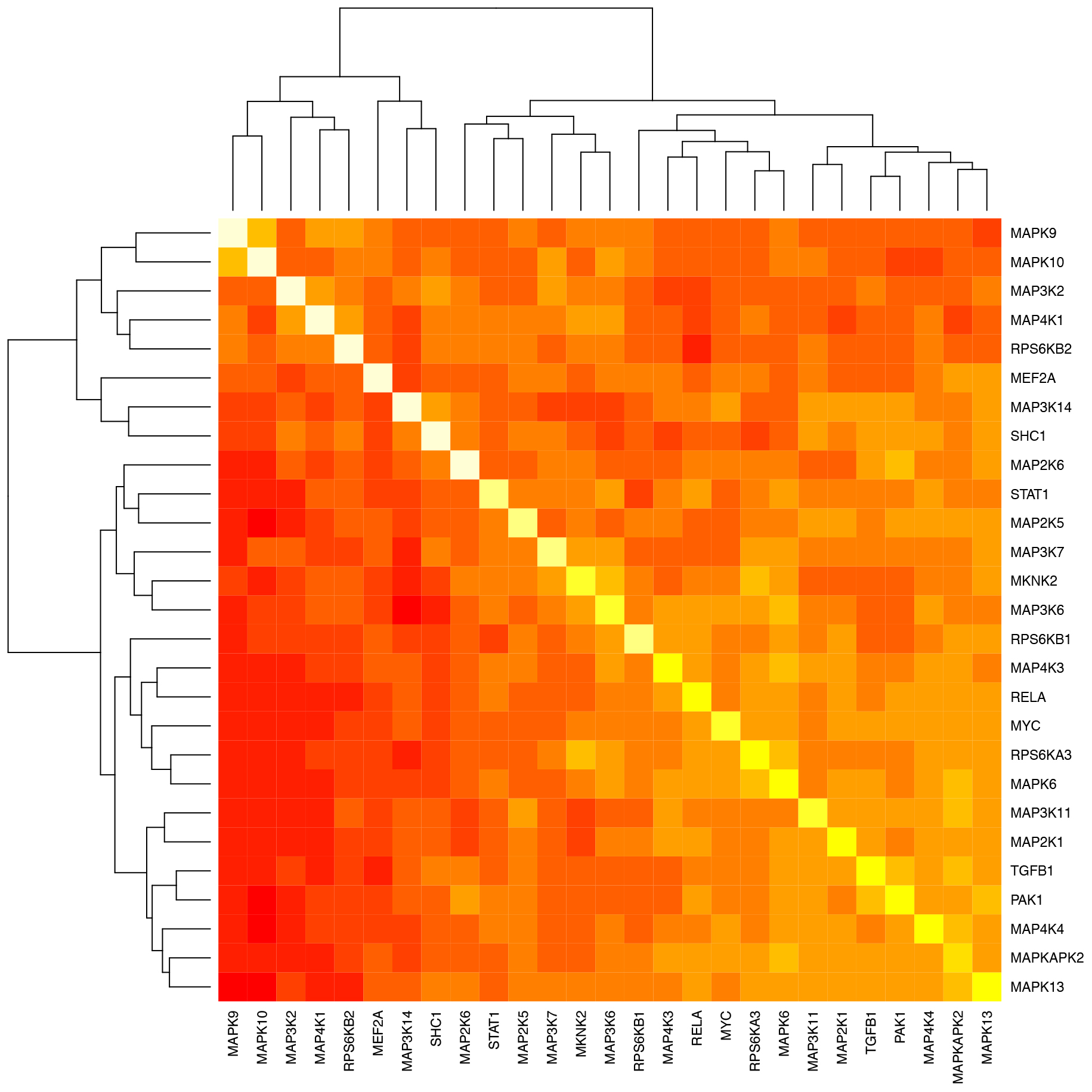

Supplement: Supplementary file 1 — Additional file 1: Clustered heatmaps of the observed correlations between our full MAPK gene set and those shown to be associated with air pollution. (A) Unadjusted DNA methylation coefficients were used to cluster the 84 genes listed in Table 1; (B) clustering of adjusted methylation coefficients, after accounting for all relevant confounders included in this study—age, blood pressure, smoking status, blood cell proportions, etc. (described in Methods); similarly, the same is shown for the 27 MAPK gene hits from Table 3, prior to adjusting for all confounders (C) and afterwards (D). (ZIP 3 MB) [file 12940_2014_810_MOESM1_ESM.zip › 10018_7989112011285506_add1/10018_7989112011285506_add1C.jpeg]

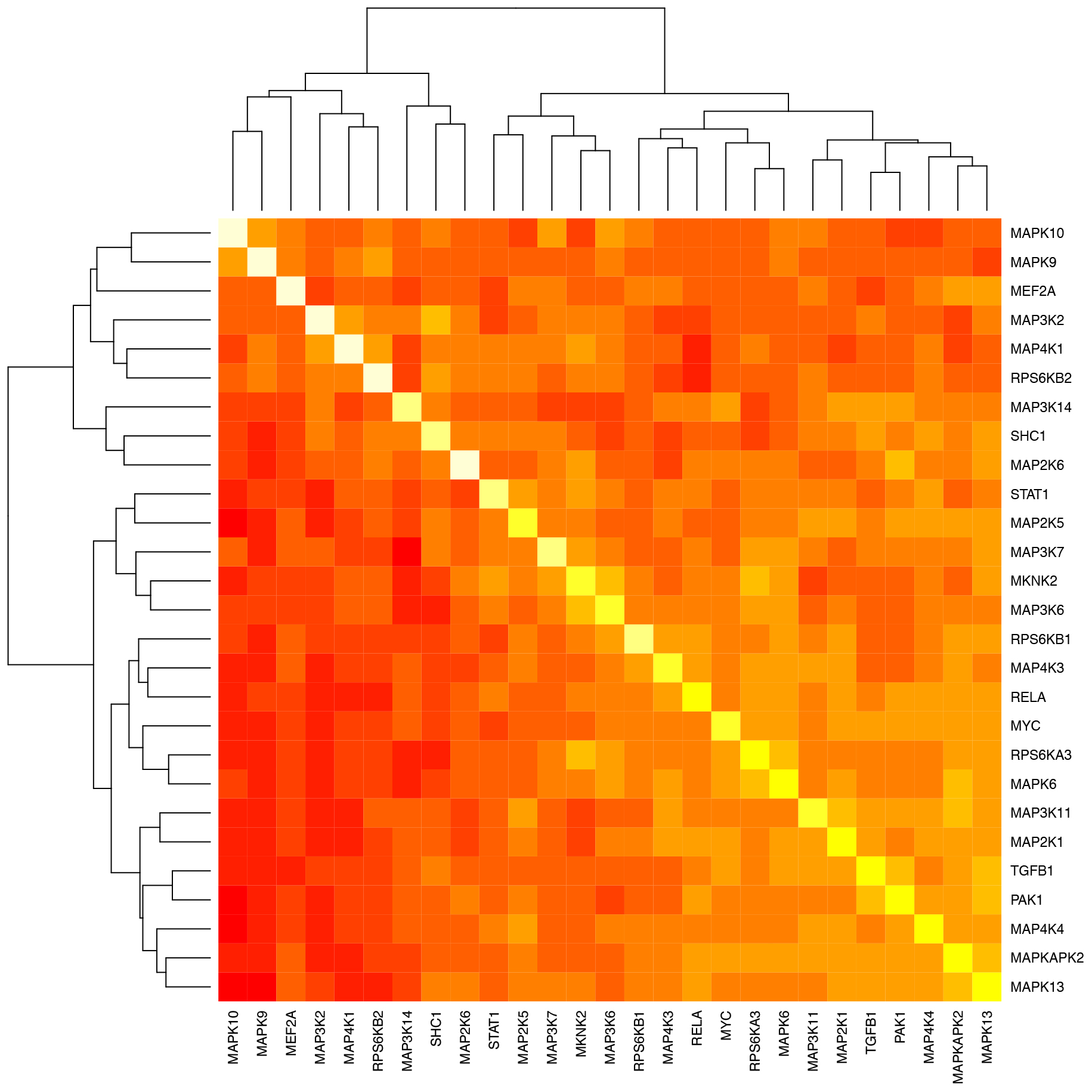

Supplement: Supplementary file 1 — Additional file 1: Clustered heatmaps of the observed correlations between our full MAPK gene set and those shown to be associated with air pollution. (A) Unadjusted DNA methylation coefficients were used to cluster the 84 genes listed in Table 1; (B) clustering of adjusted methylation coefficients, after accounting for all relevant confounders included in this study—age, blood pressure, smoking status, blood cell proportions, etc. (described in Methods); similarly, the same is shown for the 27 MAPK gene hits from Table 3, prior to adjusting for all confounders (C) and afterwards (D). (ZIP 3 MB) [file 12940_2014_810_MOESM1_ESM.zip › 10018_7989112011285506_add1/10018_7989112011285506_add1D.jpeg]
